# Ozone-induced IL-17A and neutrophilic airway inflammation is orchestrated by the caspase-1-IL-1 cascade

**DOI:** 10.1038/srep18680

**Published:** 2016-01-07

**Authors:** Luanqing Che, Yan Jin, Chao Zhang, Tianwen Lai, Hongbin Zhou, Lixia Xia, Baoping Tian, Yun Zhao, Juan Liu, Yinfang Wu, Yanping Wu, Jie Du, Wen Li, Songmin Ying, Zhihua Chen, Huahao Shen

**Affiliations:** 1 Department of Respiratory and Critical Care Medicine, Second Affiliated Hospital, Zhejiang University School of Medicine, Hangzhou, Zhejiang 310009, China; 2 Beijing Anzhen Hospital, Capital Medical University, Key Laboratory of Remodeling-Related Cardiovascular Diseases, The Ministry of Education, Beijing Institute of Heart, Lung, and Blood Vessel Diseases, Beijing 100029, China; 3 State Key Laboratory of Respiratory Diseases, Guangzhou, Guangdong 510120, China; 4 Department of Respiratory Diseases, Taizhou Municipal Hospital, Taizhou, Zhejiang 318000, China

## Abstract

Ozone is a common environmental air pollutant leading to respiratory illness. The mechanisms regulating ozone-induced airway inflammation remain poorly understood. We hypothesize that ozone-triggered inflammasome activation and interleukin (IL)-1 production regulate neutrophilic airway inflammation through IL-17A. Pulmonary neutrophilic inflammation was induced by extended (72 h) low-dose (0.7 ppm) exposure to ozone. *IL-1 receptor 1 (Il1r1)*^−/−^, *Il17a*^−/−^ mice and the caspase-1 inhibitor acetyl-YVAD-chloromethylketone (Ac-YVAD-cmk) were used for *in vivo* studies. Cellular inflammation and protein levels in bronchial alveolar lavage fluid (BALF), cytokines, and IL-17A-producing γδT-cells, as well as mitochondrial reactive oxygen species (ROS), mitochondrial DNA (mtDNA) release, and inflammasome activation in lung macrophages were analyzed. Ozone-induced neutrophilic airway inflammation, accompanied an increased production of IL-1β, IL-18, IL-17A, Granulocyte-colony stimulating factor (G-CSF), Interferon-γ inducible protein 10 (IP-10) and BALF protein in the lung. Ozone-induced IL-17A production was predominantly in γδT-cells, and *Il17a*-knockout mice exhibited reduced airway inflammation. Lung macrophages from ozone-exposed mice exhibited higher levels of mitochondrial ROS, enhanced cytosolic mtDNA, increased caspase-1 activation, and higher production of IL-1β. *Il1r1*-knockout mice or treatment with Ac-YVAD-cmk decreased the IL-17A production and subsequent airway inflammation. Taken together, we demonstrate that ozone-induced IL-17A and neutrophilic airway inflammation is orchestrated by the caspase-1-IL-1 cascade.

Asthma is a chronic airway disease characterized by airway inflammation, airway obstruction, and airway hyperresponsiveness (AHR). Patients with mild or moderate asthma provide evidence that eosinophils are the main inflammatory cells in the airway, and these patients respond very well to corticosteroid treatment. However, severe asthma patients are corticosteroid resistant. Bronchoscopic studies showed that neutrophils predominantly exist in the airways of severe asthma patients^1^. The hallmark of mild/moderate asthma is T-helper (Th)2 cytokine elevation, while the biomarkers of severe asthma are IL-1β or IL-17A-producing cells in the airway. Th17-associated cytokines IL-17A and IL-17F were reported to exist in severe asthma and were correlated with disease severity^2^.

Air pollution with ozone is common and leads to asthmatic symptoms such as airway inflammation and AHR. Moreover, established asthma patients are susceptible to ozone exposure. Ozone-induced lung inflammation is characterized by neutrophil infiltration, AHR, and the release of pro-inflammatory cytokines, such as tumor necrosis factor α (TNFα), IL-6, IL-1β, and IL-17A^3^,^4^,^5^. IL-1β was reported to play a key role in IL-17A production. IL-β synergizes with IL-6 and IL-23 to induce expression of the transcription factors interferon regulatory factor 4 and retinoic acid-related orphan receptor gamma t (*Rorγt*), thereby driving the induction of Th17 cells^6^. The secretion of IL-1β and IL-18 is controlled by the inflammatory signaling platform called ‘inflammasome’^7^,^8^,^9^, which is activated upon cellular infection or stress that triggers maturation of the above pro-inflammatory cytokines to engage innate immune defenses^7^. The inflammasome mediates the cleavage and activation of caspase-1, leading to the maturation and secretion of IL-1β and IL-18^10^. Inflammasome^11^ and IL-1 signaling^12^ have been reported in association with ozone-induced neutrophilic airway inflammation. However, the underlying mechanisms are still unclear.

In the present study, we hypothesize that ozone might activate the inflammasome to induce release of IL-1β and IL-18 in lung, and the latter in turn increases the production of IL-17A, ultimately leading to neutrophilic airway inflammation.

## Results

### Ozone induces neutrophilic airway inflammation

**Figure 1:**
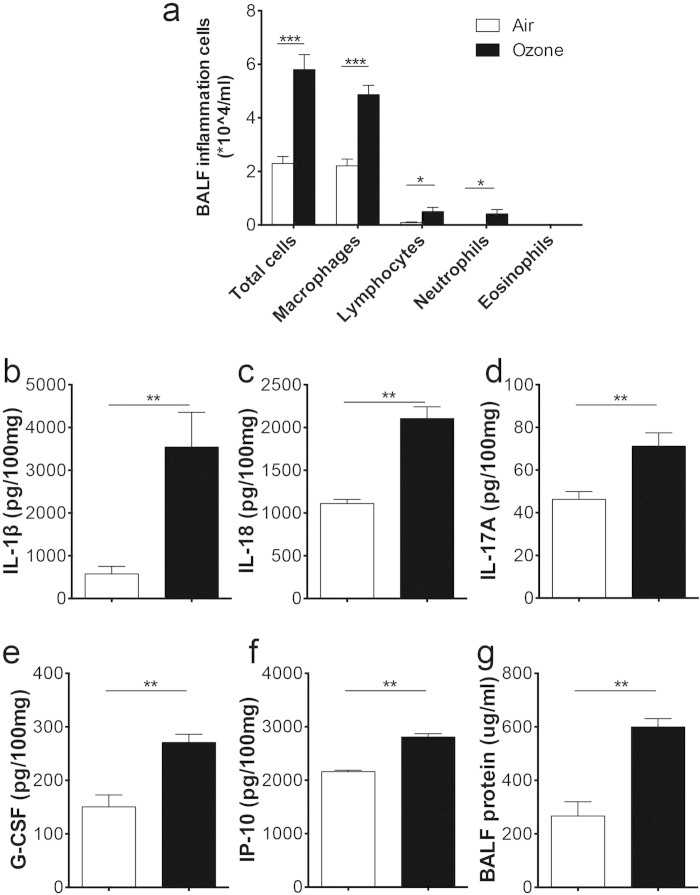
Ozone induces a neutrophilic airway inflammation. WT mice were exposed to air (white bar) or 0.7 ppm ozone for 72 h, and sacrificed 24 h later. (**a**) Total and differential cell counts of macrophages, lymphocytes, neutrophils, and eosinophils in BALF. Concentrations of IL-1β (**b**), IL-18 (**c**), IL-17A (**d**), G-CSF (**e**), and IP-10 (**f**) in lung homogenates. The cytokine concentrations were normalized to 100 mg total protein of lung homogenates. (**g**) Protein levels in BALF. Data shown were 6–8 mice per group. *p < 0.05, **p < 0.01, ***p < 0.001.

Mice were exposed to 0.7 ppm ozone or room air for 72 h and examined 24 h later. The inflammatory cell influx in BALF after exposure to ozone was increased (Fig. 1a). Next, we measured the cytokine levels in lung homogenates. The inflammasome-related cytokines IL-1β (Fig. 1b) and IL-18 (Fig. 1c) were increased in ozone-exposed mice, and the levels of IL-17A were significantly increased although at a relatively low concentration (Fig. 1d). Further analyses demonstrated that the IL-17A-driven cytokines G-CSF (Fig. 1e) and IP-10 (Fig. 1f) were increased upon ozone exposure. BALF protein levels (Fig. 1g) were also increased in the ozone-exposed mice.

### The γδT-cells contribute to IL-17A production upon ozone challenge

**Figure 2:**
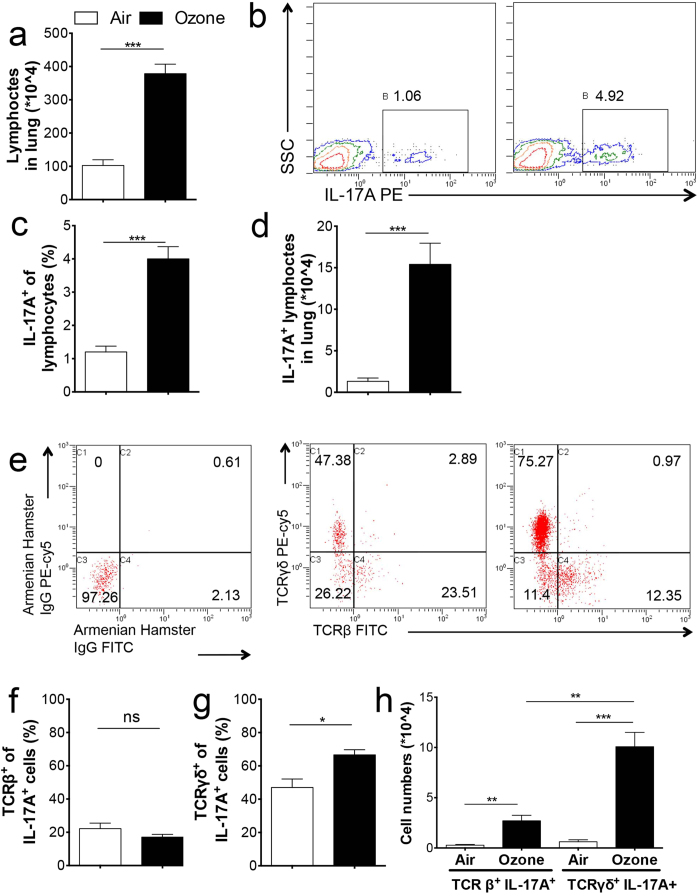
The γδT-cells contribute to IL-17A production upon ozone challenge. WT mice were exposed to air (white bar) or 0.7 ppm ozone (black bar) for 72 h, and sacrificed 24 h later. Lymphocytes were isolated, counted (**a**), gated by forward-scatter (FSC) and side-scatter (SSC), and were analyzed by staining of IL-17A (**b,c**). Representative flow cytometry profiles were shown in (**b**). (**c**) Quantification analysis from b. (**d**) IL-17A^+^ lymphocytes were calculated. The IL-17A^+^ cells were gated and stained (**e**) left: isotype; middle: air group; right: ozone group. The IL-17A^+^ cells in the air and ozone groups were gated and next analysis of TCRβ and TCRγδ. (**f,g**) Quantification of TCRβ^+^ and TCRγδ^+^ cells in IL-17A^+^ cells. (**h**) TCRβ^+^ IL-17A^+^ and TCRγδ^+^ IL-17A^+^ cells were calculated. Data shown were 6–8 mice per group. *p < 0.05, **p < 0.01, ***p < 0.001, ns, not significant.

IL-17A has been reported to play a key role in pulmonary immune defense^13^, and the sources of IL-17A in the lung are complex. We observed that the number of lymphocytes (Fig. 2a) and IL-17A-producing lymphocytes (Fig. 2b–d) were increased after ozone exposure. Since both TCRβ^+^ and TCRγδ^+^ cells are important producers of IL-17A, we examined the source of IL-17A between the two T cell subtypes. When IL-17A-producing lymphocytes were gated and analyzed for TCRβ and TCRγδ (Fig. 2e), the results showed that TCRβ^+^ (Fig. 2f) cells accounted for a low percentage, while TCRγδ^+^ cells (Fig. 2g) constituted the majority of IL-17A^+^ lymphocytes in ozone-exposed mice. Although the number of TCRβ^+^ IL-17A^+^ cells was also significantly increased upon ozone exposure, it was only about 30% of that of TCRγδ^+^ IL-17A^+^ cells (Fig. 2h).

### *Il17a*^−/−^ mice are protected from ozone-induced airway inflammation

**Figure 3:**
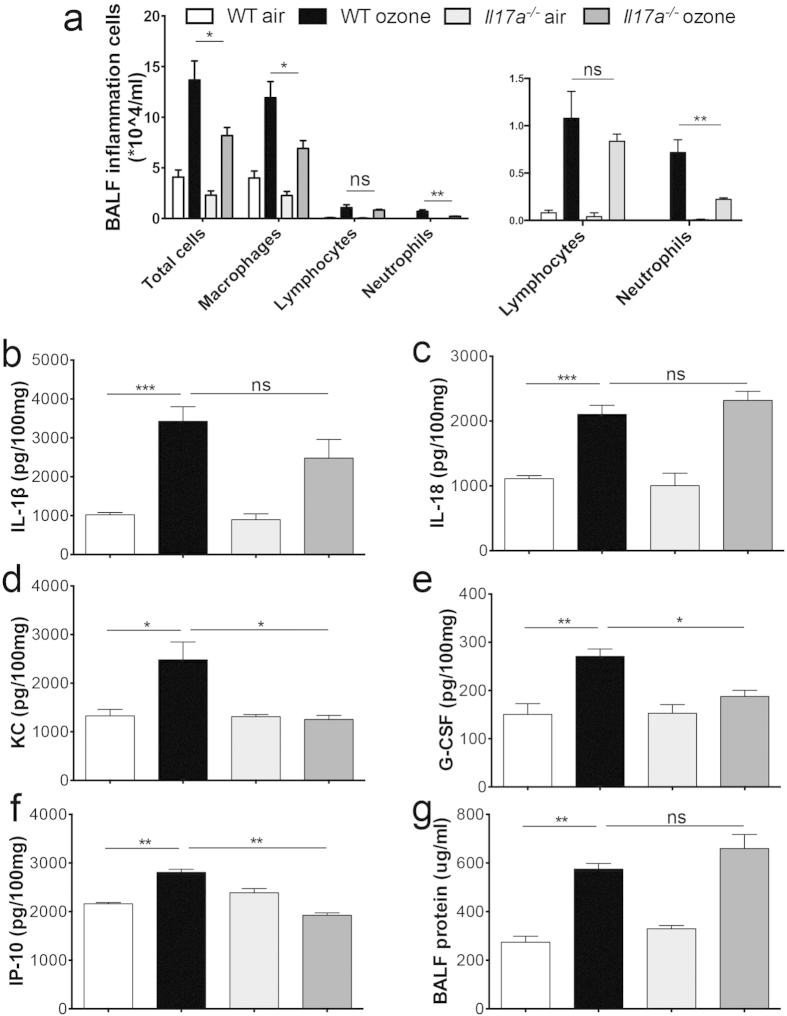
*Il17a*^−/−^ mice were protected from ozone-induced airway inflammation. WT and *Il17a*^−/−^ mice mice were exposed to air or 0.7 ppm ozone for 72 h, and sacrificed 24 h later (white bar: WT air, black bar: WT ozone, light grey bar: *Il17a*^−/−^ air, dark grey bar: *Il17a*^−/−^ ozone). (**a**) Total and differential cell counts in BALF from WT and *Il17a*^−/−^ mice exposed to ozone, with levels of lymphocytes and neutrophils shown in an enlarged scale. Cytokine and chemokine levels of IL-1β (**b**), IL-18 (**c**), KC (**d**), G-CSF (**e**) and IP-10 (**f**) measured by Elisa in lung homogenates from WT and *Il17a*^−/−^ mice. The cytokine concentrations were normalized to 100 mg total protein of lung homogenates. (**g**) Protein levels in BALF. Data shown were 6–8 mice per group. *p < 0.05, **p < 0.01, ***p < 0.001, ns, not significant.

To further demonstrate the importance of IL-17A in ozone-induced neutrophilic airway inflammation, *Il17a*^−/−^ mice were exposed to ozone for 72 h. *Il17a*^−/−^ mice showed significant reduction in airway inflammation. Compared with wild-type (WT) mice, *Il17a*^−/−^ mice had remarkably reduced numbers of total cells, macrophages, and neutrophils (Fig. 3a). The levels of the C-X-C chemokine KC (Fig. 3d) were significantly decreased in *Il17a*^−/−^ mice. The IL-17A-driven cytokines G-CSF and IP-10 were also significantly decreased in *Il17a*^−/−^ mice (Fig. e,f). However, the levels of IL-1β and IL-18 (Fig. 3b,c) and BALF protein (Fig. 3g) did not change. Two-way ANOVA indicated interactions between genetic type and exposure on neutrophils, KC, G-CSF, and IP-10.

### The IL-1 pathway is required for ozone-induced inflammation

**Figure 4:**
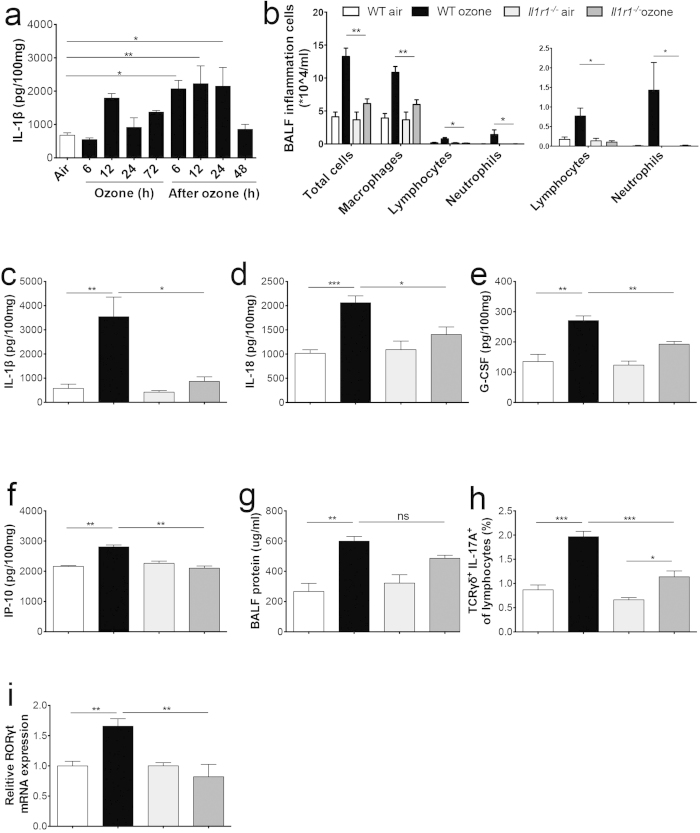
*Il1r1*^−/−^ mice failed to develop ozone-induced airway inflammation. (**a**) IL-1β levels (measured by ELISA) in lung homogenates from mice exposed to 0.7 ppm ozone over a 120-h time course. WT and *Il1r1*^−/−^ mice were exposed to air or 0.7 ppm ozone for 72 h, and sacrificed 24 h later (white bar: WT air, black bar: WT ozone, light grey bar: *Il1r1*^−/−^ air, dark grey bar: *Il1r1*^−/−^ ozone). (**b**) Total and differential cell counts of macrophages, lymphocytes, and neutrophils in BALF from WT and *Il1r1*^−/−^ mice, with levels of lymphocytes and neutrophils shown in an enlarged scale. Concentrations of IL-1β (**c**), IL-18 (**d**), G-CSF (**e**), and IP-10 (**f**) in lung homogenates from WT and *Il1r1*^−/−^ mice. The cytokine concentrations were normalized to 100 mg total protein of lung homogenates. (**g**) Protein levels in BALF. (**h**) Lymphocytes isolated from the lungs of WT and *Il1r1*^−/−^ mice were gated on FSC and SSC and stained for TCRγδ and intracellular IL-17A. Results were shown mean percentage of IL-17A-producing TCRγδ^+^ T-cells. (**i**) Relative *Rorγt* mRNA expression in lung tissues. Data shown were 6–8 mice per group. *p < 0.05, **p < 0.01, ***p < 0.001, ns, not significant.

IL-1β and IL-18 were significantly increased in the ozone-induced neutrophilic airway inflammation model (Fig. 1b,c), suggesting that the IL-1 signaling pathway might be a key regulator. Kinetic analysis of IL-1β *in vivo* following ozone exposure revealed that the IL-1β protein levels increased slowly but persisted, even at 24 h after exposure stop (Fig. 4a). It has been reported that IL-1β plays an important role in Th17 differentiation and IL-17A production^14^, so we used *Il1r1*^−/−^ mice to assess roles of the IL-1 pathway in ozone-induced pulmonary inflammation and IL-17A generation. After ozone exposure, the total cell number as well as the differential counts of neutrophils, macrophages, and lymphocytes in BALF were all significantly reduced in *Il1r1*^−/−^ mice (Fig. 4b). Ozone-induced levels of IL-1β and IL-18 were significantly reduced (Fig. 4c,d). The levels of IL-17A-driven cytokines G-CSF and IP-10 were also notably decreased in *Il1r1*^−/−^ mice (Fig. 4e,f). However, BALF protein levels were insignificantly decreased in *Il1r1*^−/−^ mice (Fig. 4g). Furthermore, the number of IL-17A^+^ γδT-cells (Fig. 4h) was significantly decreased in *Il1r1*^−/−^ mice after ozone exposure. In agreement with this, the expression of *Rorγt* induced by ozone was markedly reduced in *Il1r1*^−/−^ mice (Fig. 4i). Two-way ANOVA indicated interactions between genetic type and exposure on total cells, macrophages, lymphocytes, neutrophils, IL-1β, IL-18, G-CSF, IP-10, IL-17A^+^ γδT-cells, and relative expression of *Rorγt*.

### Ozone activates the inflammasome in lung macrophages

**Figure 5:**
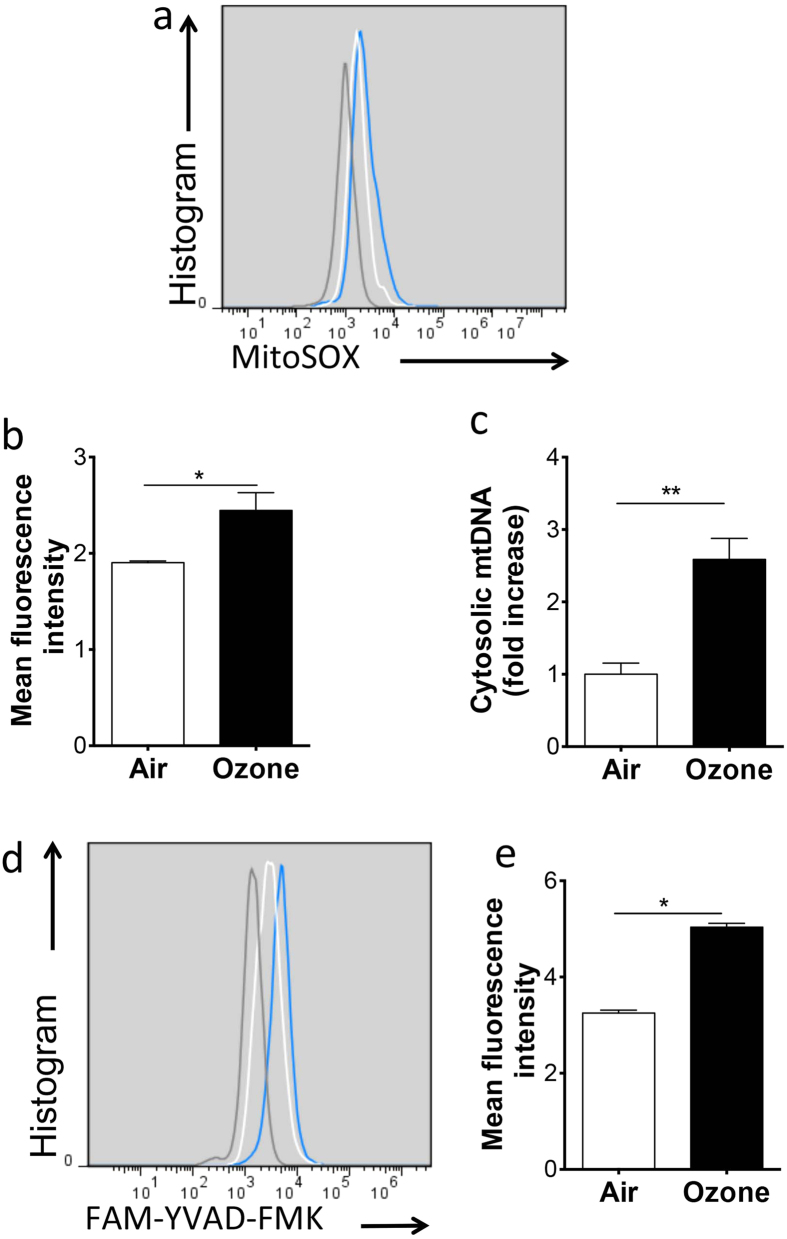
Ozone activated the inflammasome. (**a**) Macrophages isolated from lung tissues were analyzed by flow cytometry and gated by FSC and SSC. Flow cytometry analysis of lung macrophages labeled with MitoSOX from air or ozone-exposed mice, representative flow cytometry profiles (gray, unstained; white, air; blue, ozone) were shown; (**b**) Quantification of a. (**c**) Quantitative PCR analysis of cytosolic mtDNA in lung macrophages with or without ozone exposure. (**d**) Expression of active caspase-1 measured by intracellular FLICA staining of macrophages, representative flow cytometry profiles (gray, unstained; white, air; blue, ozone) were shown; (**e**) Quantification of d. Data shown were 6–8 mice per group. *p < 0.05, **p < 0.01.

*Il1r1*^−/−^ mice were protected from ozone-mediated airway inflammation, demonstrating that IL-1 pathway is necessary for IL-17A^+^ γδT-cells and neutrophilic airway inflammation. We next investigated how IL-1β was generated. Ozone is a strong oxidizer and has acute and chronic effects through an inflammatory mechanism related to oxidative stress^15^,^16^,^17^,^18^. Inflammasome is key to mediate maturation and secretion of IL-1β. Therefore, we next analyzed oxidative stress and inflammasome activation in ozone-exposed mice. Since macrophages were producers of IL-1β, we extracted lung macrophage to study the mechanisms of inflammasome activation. Flow cytometric analysis of MitoSOX demonstrated that higher levels of mitochondrial ROS were generated in lung macrophages upon ozone exposure (Fig. 5a,b). Moreover, in agreement with the reports that damage-associated mtDNA activates the inflammasome when liberated into the extracellular space^8^,^9^,^19^. We also found that ozone induced the cytosolic accumulation of mtDNA in macrophages. The relative mtDNA expression in the cytosol of macrophages isolated from ozone-exposed mouse lung was significantly increased (Fig. 5c). Notably, caspase-1 was activated in these macrophages (Fig. 5d,e).

### Inhibition of caspase-1 decreases ozone-induced airway inflammation

**Figure 6:**
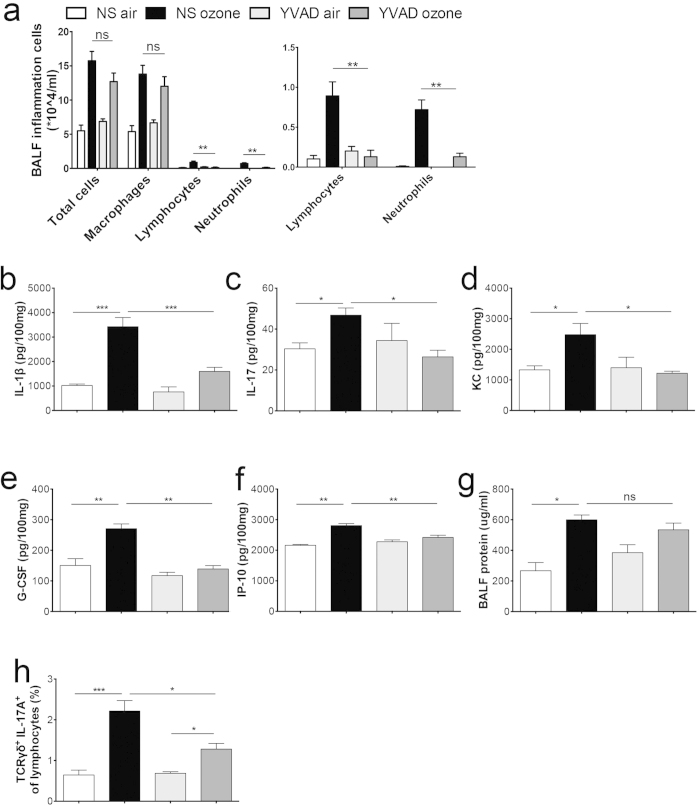
Inhibition of caspase-1 suppresses IL-17A production by γδT-cells and attenuates ozone-induced neutrophilic airway inflammation. C57BL/6 mice were exposed to ozone for 72 h, with injection of NS or the caspase-1 inhibitor Ac-YVAD-cmk (10 mg/kg) (white bar: NS air, black bar: NS ozone, light grey bar: YVAD air, dark grey bar: YVAD ozone). (**a**) Total and differential cell counts of macrophages, lymphocytes, and neutrophils in BALF, with levels of lymphocytes and neutrophils shown in an enlarged scale. Concentrations of IL-1β (**b**), IL-17A (**c**), KC (**d**), G-CSF (**e**), and IP-10 (**f**) in lung homogenates from NS- and Ac-YVAD-cmk-treated mice after ozone exposure. (**g**) Protein levels in BALF. (**h**) Lymphocytes isolated from the lungs of NS- and Ac-YVAD-cmk- treated mice were gated on FSC and SSC and stained for TCRγδ and intracellular IL-17A. Results show the mean percentage of IL-17A-producing TCRγδ^+^ T-cells. YVAD: Ac-YVAD-cmk treated group. Data shown were 6–8 mice per group. *p < 0.05, **p < 0.01, ***p < 0.001, ns, not significant.

Caspase-1 is another key regulator of inflammasome activation. Next, we used caspase-1 inhibitor to further investigate the role of caspase-1-IL-1 cascade in ozone-induced airway inflammation and in IL-17A^+^ γδT-cells generation. It has been reported that the inflammasome is required for ozone-induced airway inflammation^11^, while the detailed mechanisms remain unclear. As expected, after ozone exposure, the levels of inflammatory neutrophils and lymphocytes in BALF were significantly reduced in Ac-YVAD-cmk-injected mice (Fig. 6a). Ozone exposure failed to cause increases in IL-1β and IL-17A production in these mice (Fig. 6b,c). The levels of the C-X-C chemokine KC (Fig. 6d), as well as the IL-17A-driven cytokines G-CSF and IP-10 (Fig. 6e,f), were decreased in Ac-YVAD-cmk-injected mice, although the BALF protein levels were not decreased (Fig. 6g). Consistent with this, the number of IL-17A^+^ γδT-cells (Fig. 6h) was significantly decreased in Ac-YVAD-cmk-injected mice after exposure to ozone. Two-way ANOVA indicated interactions between exposure and treatment on lymphocytes, neutrophils, IL-1β, IL-17A, G-CSF, IP-10, KC, and IL-17A^+^ γδT-cells.

## Discussion

In this study, we demonstrate that sub-acute exposure to ozone induces a production of IL-17A and neutrophilic airway inflammation, which is mediated by a mechanism involving caspase-1-IL-1 cascade. Ozone exposure initially triggers a sequence of mitochondrial ROS generation, mtDNA release, inflammasome activation, and IL-1 production, and the latter in turn induces the IL-17A production primarily in γδT-cells, thereby driving neutrophilic airway inflammation. It is well documented that IL-17A is responsible for ozone-induced pulmonary inflammation and lung injury^5^,^20^. However, the detailed mechanisms by which IL-17A is generated remain unclear, and the results from available studies are largely inconsistent. For example, it has been reported that natural killer T-cells play a key role in ozone-induced IL-17A production and AHR^5^, while another study has demonstrated that γδT-cells are the major source of lung IL-17A after ozone exposure through the TNFα pathway^4^. Our results also demonstrate that IL-17A is essential for ozone-induced airway inflammation and that TCRγδ^+^ γδT-cells may contribute predominantly to IL-17A production. It is reasonable to conclude that the sub-acute ozone exposure transiently induces innate IL-17A production in γδT-cells, while the formation of Th cells generally represents a long-term adaptive immune response. To support this, IL-17A-producing γδT-cells are also found in diseases associated with innate immunity as well as autoimmune diseases^21^,^22^. TCRβ^+^ TCRγδ^−^ cells appear to play a less important role in our model, since ozone exposure only induces a small percentage of these cells. However, the eventual role of other cells such as NK cells or NKT cells in the production of IL-17A cannot be ruled out completely.

In our study, the ozone-induced IL-17A-producing γδT-cells were apparently regulated by the inflammasome and subsequent IL-1 signaling, as either blockade of IL-1 by *Il1r1*-knockout or caspase-1 inhibitor effectively decreased the levels of IL-17A-producing γδT-cells as well as the consequent neutrophilic inflammation. In fact, IL-1β secretion has been shown to be responsible for the innate T-cell response and upregulation of γδT-cells^23^,^24^ in autoimmune diseases. In models of ozone-induced airway inflammation, two studies have demonstrated that IL-1R1 is required for pulmonary inflammatory responses^12^,^25^. Similarly, another report has demonstrated that IL-1β is required for epidermal T-cells in the context of hypersensitivity^26^. Our current study is not only in complete agreement with the findings showing that blocking IL-1 signaling effectively attenuates ozone-induced airway inflammation, but further provides a mechanistic link demonstrating that IL-17A-producing γδT-cells bridge IL-1 signaling and airway inflammation.

A number of pathways and molecules can activate the inflammasome, most likely depending on the various cell types or stimuli. ROS are classical activators of the inflammasome, and ozone exposure evidently induces ROS. Specifically, we found that ozone exposure induced mitochondrial ROS production in lung macrophages, in agreement with a previous report^27^,^28^,^29^. Enhanced mitochondrial ROS indicates increased damage of mitochondria, which may lead to the release of mtDNA into the cytosol, thereby activating the inflammasome^10^. We also found increased mtDNA in the cytosol of macrophages exposed to ozone, suggesting a mechanism by which ozone activates the inflammasome. In fact, mitochondrial dysfunction has been reported to be responsible for severe symptoms in airway inflammation^30^ and γδT-cell up-regulation^31^, further supporting our conclusions. Recently, it has been reported that hyaluronan contributes to ozone-induced activation of the inflammasome and AHR^11^,^32^. However, it remains unclear whether there are any crosslink between the hyaluronan and mitochondrial ROS pathways.

In conclusion, here we unravel a detailed signaling pathway, where ozone-induced neutrophilic airway inflammation and IL-17A production are mediated through the mitochondrial ROS-caspase-1-IL-1 cascade. This may provide new therapeutic targets for the treatment of neutrophilic airway inflammation.

## Materials and Methods

### Mice

Female wild-type mice (WT, C57BL/6 background, 6-8 weeks old) were purchased from the Animal Center of Zhejiang University and housed in a conventional animal facility. *Il1r1*^−/−^ mice (C57BL/6 background) were purchased from the Jackson Laboratories (Bar Harbor, ME). *Il17a*^−/−^ mice (C57BL/6 background) were purchased from the Center for Experimental Medicine and Systems Biology (Institute of Medical Science, University of Tokyo, Japan). Mice were housed in a room maintained at 23+/–2 °C with 50%+/–10% humidity and a 12-h light: 12-h dark cycle (lights on from 8:00 a.m. to 8:00 p.m.) In addition, they were allowed free access to water and regular rodent chow. Mice were anesthetized with pentobarbital sodium before sacrifice. All the animal experiments were strictly conducted in accordance with the protocols approved by the Ethics Committee for Animal Studies at Zhejiang University, China.

### Ozone exposure

Mice received a sub-acute (72 h) ozone exposure at 0.7 ppm from an ozone generator (LS-F9, Laisen). Cages containing conscious mice were placed inside a stainless-steel and Plexiglas chamber with a high-efficiency air supply, where they were exposed to ozone. During exposure, the animals had continuous access to food and water. Ozone concentrations within the chamber were maintained by adjusting the flow rates of both room air and ozone. The concentrations of ozone were continuously monitored (HD5+ Ozone, Huideng). Control mice were exposed to room air.

### Treatment with caspase-1 inhibitor

The caspase-1 inhibitor Ac-YVAD-cmk (Sigma Aldrich) was dissolved in normal saline (NS) and administered intraperitoneally (i.p.) at 10 mg/kg 30 minutes before ozone exposure. Control mice were injected with NS.

### BALF analysis

After euthanasia, the left lung was lavaged 3 times each with 0.4 ml PBS. We performed lavage analysis to determine differential cell counts as previously described^33^. The right lung was snap-frozen in liquid nitrogen for homogenates.

### Lung homogenates

Lung tissues were homogenized in Radio Immunoprecipitation Assay (RIPA) Lysis Buffer (Beyotime) and then centrifuged at 12,000 relative centrifugal force (rcf) for 20 min. Total protein levels in the homogenates were measured using a Pierce BCA protein assay kit (Thermo).

### Isolation of immune cells from lung

Lung tissues of mice were digested with collagenase and minced. The isolated cells were underlain with 3 ml lymphocyte separation solution (DAKEWE), centrifuged at 800 rcf for 30 min. The isolated cells were incubated for 2 h and adherent cells were harvested as lung macrophages. The cells suspended in the culture medium were harvested as lung lymphocytes.

### Flow cytometric analysis

Lymphocytes isolated from the lung were cultured in 1640 medium and 10% FBS. Before FACS analysis, cells were stimulated with 25 ng/mL phorbol 12-myristate 13-acetate (PMA, ebioscience), 1ug/mL ionomycin (ebioscience), and Brefeldin A (BFA ebioscience) at 37 °C for 4 hours. Isolated lymphocytes were stained with FITC-conjugated anti-mouse TCRβ (clone H57-597, eBioscience), and PE-cy5-conjugated anti-mouse TCRγδ (clone eBioGL3, eBioscience). After surface staining, cells were washed, fixed, and permeabilized for intracellular staining with PE-conjugated anti-mouse IL-17A (clone eBio17B7, eBioscience). All fluorophore-conjugated antibodies and the corresponding isotypes were from eBioscience. The stained cells were used for flow cytometric analysis (FC500, Beckman).

### Measurement of mtDNA in macrophage cytosol

Lung macrophages were isolated and homogenized in 100 mM tricine-NaOH solution, pH 7.4, containing 0.25 M sucrose, 1 mM EDTA, and protease inhibitor, then centrifuged at 700 rcf for 10 min at 4 °C. Protein concentrations and supernatant volumes were normalized, followed by centrifugation at 10,000 rcf for 30 min at 4 °C for the production of supernatant corresponding to the cytosolic fraction^10^. Total DNA was isolated with a DNeasy Blood & Tissue kit (Qiagen). Quantitative PCR was used to measure mtDNA with SYBR Green PCR Master Mix (Takara) and established primers for mitochondrial and nuclear genes. The relative expression of mtDNA was normalized to that of nuclear DNA as the ratio of DNA encoding cytochrome c oxidase I to nuclear DNA encoding 18S ribosomal RNA^10^. The relative difference in mtDNA levels was calculated by 2^−ΔΔCt^ methods. The following primers were used:

18S forward, 5-TAGAGGGACAAGTGGCGTTC-3;

18S reverse, 5-CGCTGAGCCAGTCAGTGT-3;

mouse cytochrome c oxidase I forward, 5-GCCCCAGATATAGCATTCCC-3;

mouse cytochrome c oxidase I reverse, 5-GTTCATCCTGTTCCTGCTCC-3.

### RNA isolation and Quantitative Real Time PCR analysis

RNA from lung was isolated using Trizol (Invitrogen). Reverse transcription was performed with Reverse Transcription Reagents (TaKaRa). The relative expressions of mouse *Rorγt* were normalized to *Actb* levels. The relative difference in mRNA levels was calculated by 2^−ΔΔCt^ methods. All protocols were performed according to the manufacturer’s instructions. The following primers were used:

*Actb* Forward: 5- GGCTGTATTCCCCTCCATCG-3;

*Actb* Reverse: 5- CCAGTTGGTAACAATGCCATGT-3;

*Rorγt* Forward: 5- CGCGGAGCAGACACACTTA-3;

*Rorγt* Reverse: 5- CCCTGGACCTCTGTTTTGGC-3.

### MitoSOX

Mitochondrial ROS was measured in cells by MitoSOX (Invitrogen) staining (5 μM for 15 min at 37 °C)^34^ and analyzed by flow cytometry.

### Caspase-1 Detection

Activation of the caspase-1 inflammasome in macrophages was detected by FAM-FLICA caspase-1 Assay Kit (Immunochemistry Technologies) according to the manufacturer’s protocol.

### ELISA analysis

Cytokine levels in lung homogenates and cell culture supernatants were analyzed by ELISA using paired antibodies (R&D Systems) following the manufacturer’s instructions.

### Statistical analysis

Results were presented as mean ± standard error of the mean. Student’s t-test (two-tailed) was used to for cooperation between groups. We applied two-way analysis of variance (ANOVA) to determine the interactions between exposure and genotype/treatment on BALF cells, cytokines, T cells and mRNA expression. For comparing these indexes of each group, Tukey post hoc tests were performed. Differences were considered statistically significant if p < 0.05.

## Additional Information

**How to cite this article**: Che, L. *et al*. Ozone-induced IL-17A and neutrophilic airway inflammation is orchestrated by the caspase-1-IL-1 cascade. *Sci. Rep*. **6**, 18680; doi: 10.1038/srep18680 (2016).
